# Enhanced Visible-Light-Responsive Photocatalytic Degradation of Ciprofloxacin by the Cu_x_O/Metal-Organic Framework Hybrid Nanocomposite

**DOI:** 10.3390/nano13020282

**Published:** 2023-01-09

**Authors:** Cheng-Kuo Tsai, Ching-Hsuan Huang, Jao-Jia Horng, Hui Lin Ong, Ruey-An Doong

**Affiliations:** 1 Department of Safety Health and Environmental Engineering, National Yunlin University of Science and Technology, Douliou 64002, Taiwan; 2 Emergency Response Information Center, National Yunlin University of Science and Technology, Douliou 64002, Taiwan; 3 Institute of Environmental Engineering, National Yang Ming Chiao Tung University, Hsinchu 30010, Taiwan; 4 Faculty of Chemical Engineering Technology, Universiti Malaysia Perlis, Arau 02600, Malaysia; 5 Centre of Excellence for Biomass Utilization and Taiwan-Malaysia Innovation Centre for Clean Water and Sustainable Energy (WISE Centre), Universiti Malaysia Perlis (UniMAP), Jejawi 02600, Malaysia; 6 Institute of Analytical and Environmental Sciences, National Tsing Hua University, Hsinchu 30013, Taiwan

**Keywords:** copper metal organic framework, Cu_x_O/MOF, adsorption, photodegradation, ciprofloxacin

## Abstract

Ciprofloxacin (CIP) is a commonly used antibiotic, however, once in the environment, it is highly toxic with a poor biodegradability. Given these attributes, an effective strategy for the removal of CIP is urgently needed for the protection of water resources. Herein, a novel copper metal-organic framework (Cu_x_O/MOF) multifunctional material has been produced, in this work, by the calcination of Cu-MOF urea at 300 °C, in the presence of a 5% H_2_ atmosphere. The morphological, structural, and thermal properties of the prepared Cu_x_O/MOF were determined through various techniques, and its photocatalytic behavior was investigated for the degradation of CIP under visible-light irradiation. The prepared Cu_x_O/MOF bifunctional material is presented as a graphitic carbon-layered structure with a particle size of 9.2 ± 2.1 nm. The existence of CuO-Cu_2_O-C, which was found on the Cu_x_O/MOF surface, enhanced the adsorption efficiency and increased the photosensitivity of Cu_x_O/MOF, towards the degradation of CIP in aqueous solutions. The tailored Cu_x_O/MOF, not only shows an excellent CIP degradation efficiency of up to 92% with a constant kinetic rate (k_obs_) of 0.048 min^−1^ under visible light, but it can also retain the stable photodegradation efficiency of >85%, for at least six cycles. In addition, Cu_x_O/MOF has an excellent adsorption capacity at pH 6.0 of the maximum Langmuir adsorption capacity of 34.5 mg g^−1^ for CIP. The results obtained in this study demonstrate that Cu_x_O/MOF is a reliable integrated material and serves as an adsorbent and photocatalyst, which can open a new pathway for the preparation of visible-light-responsive photocatalysts, for the removal of antibiotics and other emerging pollutants.

## 1. Introduction

Ciprofloxacin (CIP) is commonly used as an antibiotic in the pharmaceutical industry and is found in various healthcare products, including personal care items, animal husbandry, and fish farming. The extensive usage and poor biodegradability has made CIP a widespread pollutant in surface water and groundwater resources [1,2]. High concentrations of CIP, from 150 ug L^−1^ up to 31 mg L^−1^, have been reported in effluent from hospitals and pharmaceutical factories, respectively [3]. CIP is classified as an emerging contaminant of concern because of its contribution to ecological problems and its toxicity to aquatic organisms and humans [4]. The declining water quality worldwide and subsequent difficulty to remove contaminants of concern has become a growing research need. Over the past decades, several physical, chemical, and biological techniques have been developed to remove CIP from aqueous solutions, including adsorption, biodegradation, electrochemical oxidation, and photocatalysis [5,6,7]. However, CIP has a stable chemical structure with various ion species at different pH conditions, which makes removing it from the water matrix using a monofunctional treatment technique difficult. Therefore, developing a novel and highly effective multifunctional technique for degrading CIP in water is urgently needed for the protection of water resources.

Metal-organic framework (MOF) nanomaterials, such as MOF-PNIPAM membranes [8], ZIF-8 MOF [9], PCL/MOF-545 [10], HKUST-1 [11], and ZIF-8 [12], are widely used for drug delivery, diagnosis [13,14], as adsorbents [15], and for photocatalysis, for the removal of sulfonamide, CIP, and tetracycline antibiotics, respectively, from an aqueous solution through the adsorption technique. In addition, MOFs have attracted significant attention in photocatalysis technology, due to their semiconductor-like behavior, which transfers photogenerated charges to the reactive sites of a ligand-to-metal charge transfer (LMCT) for the in-situ decomposition of organic compounds, to form CO_2_ and water [16]. Yu et al. reported that a high organic pollutant (4-nitrophenol) degradation efficiency and the total organic carbon (TOC) removal are achievable using a metal-organic framework (MOF), MIL-88-Fe, under photocatalytic ozonation [17]. The limitation is that MOFs have a reduced performance under solar light irradiation, due to their lack of visible light response. This drawback can be overcome by utilizing cuprous oxide (Cu_2_O) which can be excited in the visible light region, and it has been widely used in photocatalytic applications [18]. Meanwhile, less attention has been given in developing cuprous oxide-based bifunctional nanocomposite using an in-situ approach to enhance the CIP degradation efficiency through adsorption and photocatalysis methods.

Cuprous oxide (Cu_2_O)-based heterojunctions have attracted researchers’ attention because of their ability to capture and transfer the photogenerated electrons efficiently, to enhance the photodegradation capacity of organic subtracts under visible light [19,20,21] When the Cu active species are in the form of Cu_2_O-CuO or Cu_2_O-Cu-CuO, they function as a p-type semiconductor that can achieve photo-activity with a narrow band gap of about 2.0 eV. Where, electrons can rapidly reduce the Cu_2_O to Cu, rather than the degradation of the organic subtracts [19,22,23]. Li et al. fabricated a Cu-MOF-74-derived Cu-Cu_2_O-C nanocomposite under a nitrogen atmosphere, at 1000 °C, that effectively reduced the CO_2_ to CO under visible light [24]. This study tests the suitability of CuxO/MOF for the CIP photodegradation under controlled conditions, to understand the potential suitability for treating CIP in effluent. However, limited research has been carried out on synthesizing the multi-functional Cu-MOF-derived CuO-Cu_2_O-C nanomaterial at low temperatures, using a green approach, for the CIP removal in an aqueous solution. This could help in saving energy and lead to environmental sustainability.

**Scheme 1:**
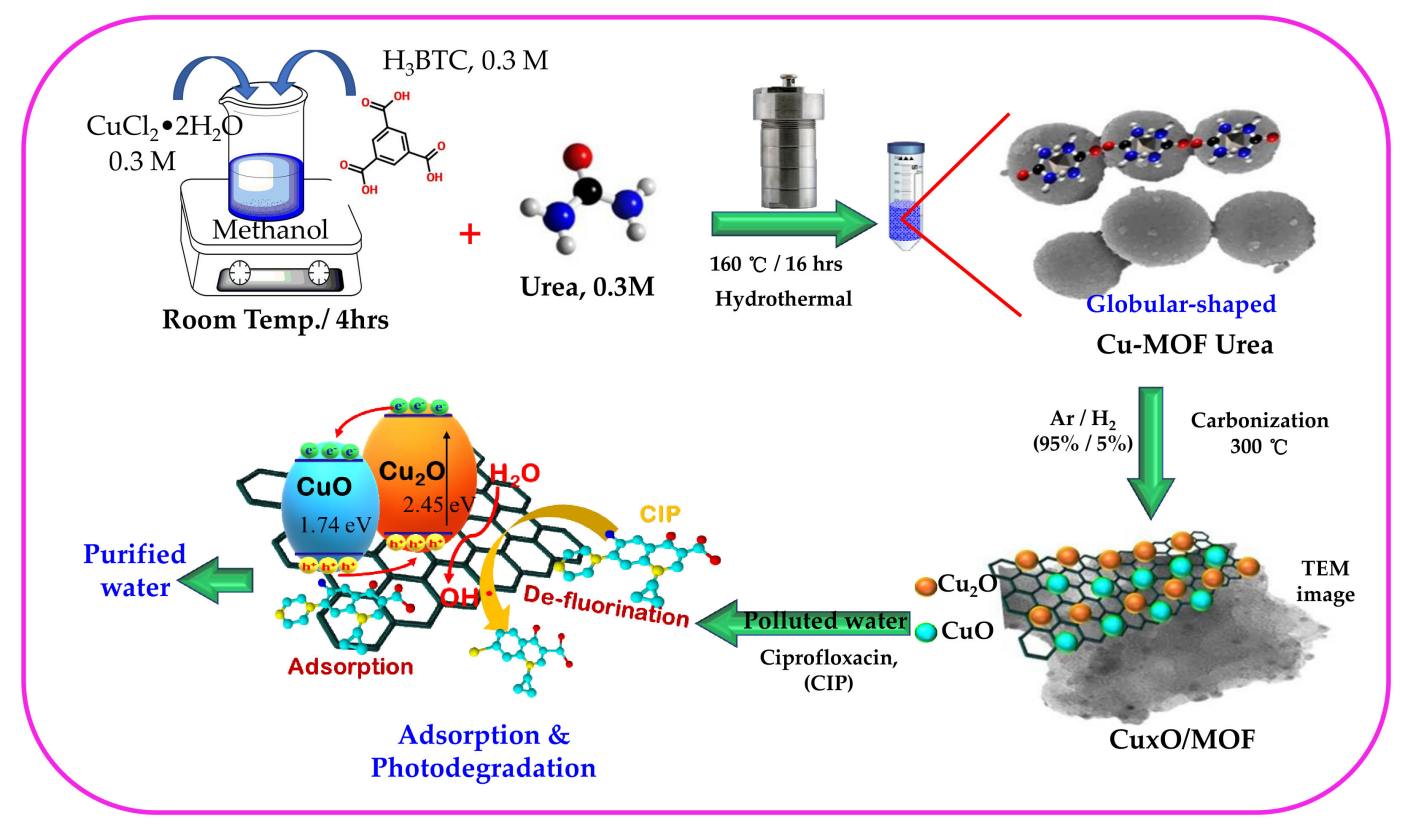
Illustration of the photo-activation of Cu_x_O/MOF for the adsorption and photodegradation of ciprofloxacin.

Urea has a high nitrogen content (N%, 46) with hydrocarbon, and it has been widely used in as a hydrogen bond-donating catalyst [25]. It demonstrates a low decomposition temperature of below 300 °C [26] and can perform the CuO reduction to Cu_2_O at a low temperature, leading to energy-saving benefits. In this work, we have successfully fabricated a bifunctional Cu_x_O/MOF nanocomposite material, in the presence of urea, for the photodegradation of CIP under visible light in an aqueous solution. As shown in Scheme 1, the Cu_x_O/MOF consists of a carbon structure with CuO-Cu_2_O nanoparticles to form a type II semiconductor heterojunction that could enhance the CIP removal efficiency. The nanoparticles of 9.2 ± 2.1 nm Cu_x_O-MOF are directly reduced through Cu-MOF-urea in a hydrogen atmosphere, at a low temperature of 300 °C for 5 h. In addition, using X-ray absorption spectroscopy (XAS), the surface structure of CuO and Cu_2_O of Cu_x_O/MOF is observed, after calcination in the H_2_ atmosphere. Herein, this Cu_x_O/MOF photocatalyst exhibits a high photodegradation performance. It is a reliable integrated material and serves as an adsorbent and photocatalyst, potentially establishing a new pathway for the development of visible-light-responsive photocatalysts for the removal of antibiotics and other emerging pollutants.

## 2. Experiment

### 2.1. Chemicals

Copper (II) chloride dihydrate (CuCl_2_ · 2H_2_O, 99%) and phosphoric acid (85%) were purchased from Sigma-Aldrich. Trimesic acid (1,3,5-benzenetricarboxylic acid (BTC), 98%) was obtained from Alfa Aesar. Urea (>99%), methanol anhydrous (99.9%), and acetonitrile (99.9%) were purchased from J. T. Baker. Deionized water (DI water, Millipore Co., Burlington, MA, USA, 18.2 MΩ cm) was used for the preparation of the chemical solutions.

### 2.2. Synthesis of Cu-MOF

Cu-MOF was fabricated by dissolving 0.3 M BTC into 15 mL of anhydrous methanol, and then stirred for 10 min under ambient conditions at 25 °C. Following the addition of 0.3 M copper (II) chloride dihydrate in 10 mL of anhydrous methanol, the dropwise addition of the mentioned solution into the BTC solution was performed using a syringe. Then, the Cu-MOF colloidal solution was again stirred for an hour, and then poured into an autoclave tube for the hydrothermal treatment at 160 °C for 16 h. Once cooled to room temperature, the resulting blue precipitate was washed with methanol several times and dried in a vacuum oven at 60 °C to obtain a light blue color of Cu-MOF.

### 2.3. Synthesis of Cu-MOF-Urea and Cu_x_O/MOF

For the preparation of the urea-coated Cu-MOF, 0.3 M of urea was well-dissolved in 10 mL of anhydrous methanol at room temperature, through magnetic stirring. Then, the urea solution was slowly amended into the Cu-MOF precursor with continuous stirring, for an hour. The mixture was then transferred into an autoclave tube for the hydrothermal preparation, at 160 °C for 16 h. Once cooled, washed, and dried, the light blue-colored Cu-MOF-urea was harvested.

The Cu_x_O/MOF hybrid nanomaterial was prepared using the carbonization method, by placing Cu-MOF-urea in a tube furnace, and then heated at 300 °C for 5 h, at a rate of 5 °C min^−1^ in 5% H_2_/Ar atmosphere.

### 2.4. Characterization of the As-Prepared MOF-Based Nanomaterials

The Cu_x_O/MOF materials were analyzed using a field emission scanning electron microscope (JOEL JSM-7800F, FE-SEM), to observe the material’s morphology. A field emission transmission electron microscope (FE-TEM, JEM 1400), at an accelerating voltage of 120 kV, was used to identify the histogram of the Cu_x_O particle size. The crystallinity of the as-prepared materials was determined using a Bruker D8 X-ray diffractometer (XRD) with Cu Ka radiation (λ = 1.5405 Å), at a voltage and current density of 40 kV and 40 mA. The chemical elemental binding states of the as-prepared materials were then analyzed with an X-ray photoelectron spectrometer (XPS, Physical Electronics, Eden Prairie, MN), using an Al Kα monochromator X-ray source, and calibrated using carbon (C 1s = 284.6 eV). The X-ray absorption spectroscopy (XAS) measurement for the Cu, C, and O K-edge, was performed at a soft X-ray beamline BL 16A located at the National Synchrotron Radiation Research Centre, Hsinchu, Taiwan. The photon energies were calibrated to an accuracy of 0.1 eV, using the Cu L-edge absorption of 931.2 eV and O K-edge transition at 530.1 eV, using CuO as a reference. The Brunauer–Emmett–Teller method was employed to determine the specific surface area and the pore structures of the photocatalysts, using an ASAP 2020 porosimetry system at 77 K. The thermal properties of the as-prepared Cu-MOF were determined with a thermogravimetric analyzer (TGA) (Mettler Toledo DSC/TGA 3+ Star system). The electron paramagnetic resonance (EPR) signals were obtained from an electron paramagnetic resonance spectrometer (E580, Bruker, Leipzig, Germany). The in situ EPR analyses, using 5,5-dimethyl-1-pyrroline-N-oxide (DMPO) with the spin trap, were performed and they identified the generation of ^•^OH radicals in an aqueous solution and ^•^O_2_^−^ in methanol, during the visible light illumination. The bandgap of the as-prepared materials was determined using the diffuse reflectance mode on a HITACHI U-4100 controller, with a U.V. solution 2.1 (HITACHI, Tokyo, Japan).

### 2.5. Photodegradation of CIP

The photodegradation of CIP over Cu_x_O/MOF, was conducted via batch experiments, using a hollow cylindrical reactor under visible light irradiation (λ = 465 ± 40 nm). One g L^−1^ of the MOF-based nanomaterials, including Cu-MOF, Cu-MOF-urea, and Cu_x_O/MOF, were added to a solution containing 10 mg L^−1^ CIP at pH 6 and at 25 °C. To further understand the adsorption behavior of CIP with Cu_x_O/MOF, the CIP solution was added to the mixture, in order to obtain the CIP concentrations between 5 and 40 mg L^−1^. Prior to the irradiation, the suspension was magnetically stirred in the dark for 60 min, to reach the desorption-desorption equilibrium. Then, the resulting suspension was exposed to the visible light for 120 min. Five mL of the CIP solution with a catalyst, was collected and allowed to pass through a 0.45 um polyvinylidene (PVDF) membrane. The initial and final concentrations of CIP were analyzed using a high-performance liquid chromatography instrument (HPLC, DIONEX UltiMate 3000) equipped with a C18 column (LUNA 5u 100A, 4.6 mm × 250 mm, Phenomenex). The CIP photodegradation products were investigated using a time of flight (TOF) liquid-chromatography mass spectrometer (LC-MS) (JMS-T100LP AccuTOFLC-plus 4G (JEOL)) with an electron spray ionization (ESI^+^) source. The effect on the CIP initial and final concentrations, and the photodegradation process were investigated. To determine the reusability of Cu_x_O/MOF, the above mentioned photodegradation experiment in six successive cycles was carried out, by adding 0.5 g L^−1^ of Cu_x_O/MOF with 15 mg L^−1^ of the CIP solution under agitation, for 120 min.

## 3. Results and Discussion

### 3.1. Surface Characterization of Cu_x_O/MOF

**Figure 1:**
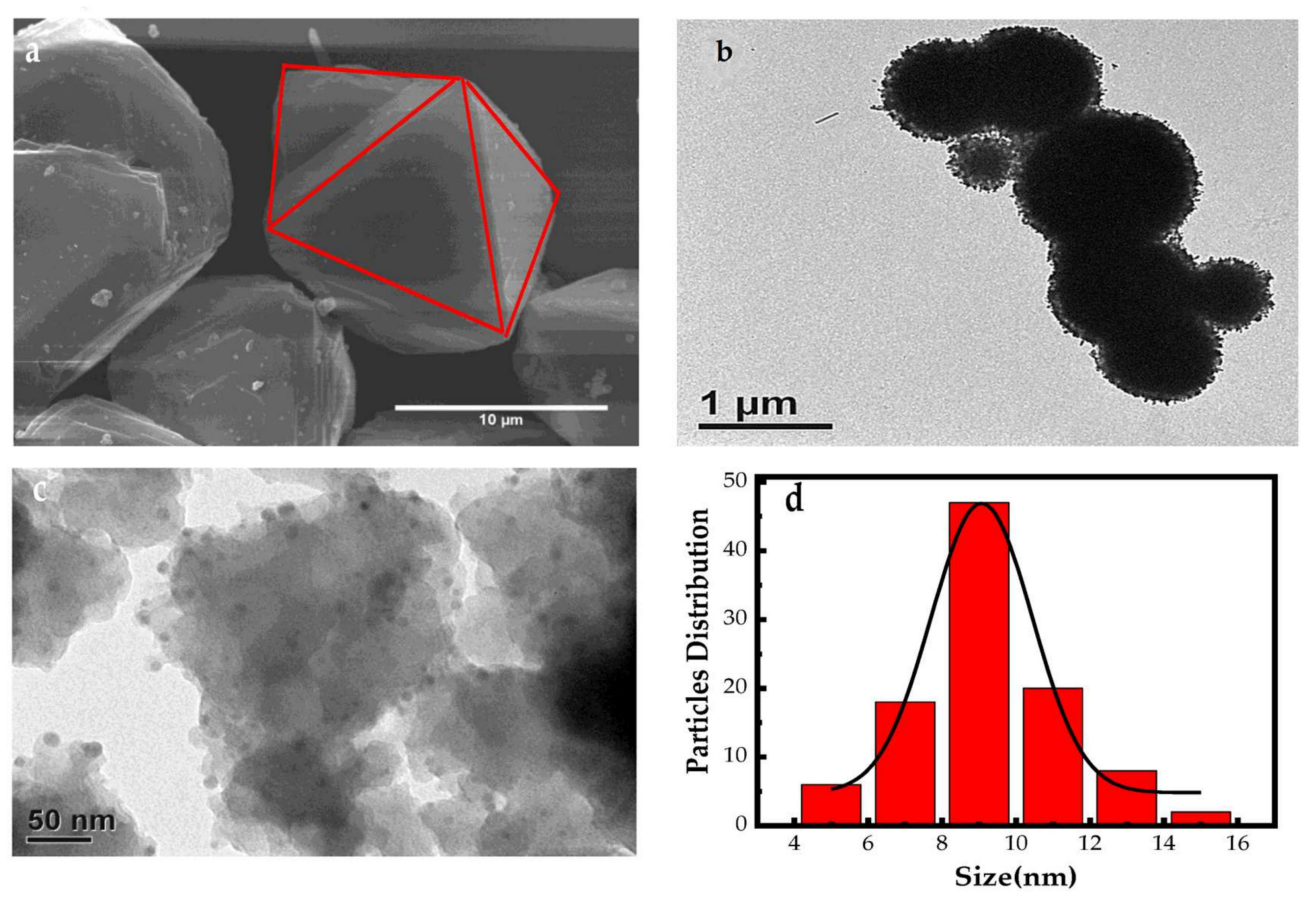
(**a**) SEM images of Cu-MOF; TEM images of (**b**) Cu-MOF-urea and (**c**) Cu_x_O/MOF, and (**d**) histogram of the Cu_x_O nanoparticles onto MOF.

The surface morphological characteristics of Cu-MOF, Cu-MOF-urea, and Cu_x_O/MOF were first examined. Figure 1a shows the octahedral structure of the Cu-MOF crystal with a pyramid-like shape, which is in good agreement with those of Cu-MOF [27]. In addition, the particle size of Cu-MOF obtained in this study, are in the range of 10–18 μm. Following the hydrothermal treatment with urea at 160 °C, the reducing power of urea decomposes the bulky pyramid shape of Cu-MOF, changing the morphology to microcapsules with a particle size of 1–2 μm (Figure S1, Supplementary Data). The transmission electron microscope (TEM) image in Figure 1b clearly shows the globular-shaped nanoparticles, that then finally are assembled into a microspherical structure. Then, the morphology of the Cu-MOF-urea materials changes significantly from the microspheres into small irregularly triangle-like nanomaterials with some nanoparticles on the surface after calcination at 300 °C in an Ar/H_2_ atmosphere (Figure S2, Supplementary Data). The TEM image of Cu_x_O/MOF clearly indicates the deposition of nanoparticles onto the surface of the irregularly shaped MOF structure (Figure 1c). This phenomenon is mainly attributed to the thermal decomposition of the O-containing substance during carbonization, resulting in the collapse of the C-skeleton [28]. During the carbonization process, the Cu ions in the retained carbon membrane structure of Cu-MOF-urea, would be oxidized to Cu_x_O nanoparticles, and subsequently deposited onto the MOF surface. In addition, the Cu_x_O nanoparticles show a narrow particle size distribution. As illustrated in Figure 1d, the histogram of the Cu_x_O nanoparticles exhibits the Gaussian distribution with an average lateral size of 9.2 ± 2.1 nm (n = 102), which is observed in the TEM image in Figure 1c. Remarkably, these nanoparticles are uniform in size and well distributed well on the carbon sheet after carbonization at 300 °C in an Ar/H_2_ atmosphere. These uniformly dispersed nanoparticles can serve as the electron trapping center, which can provide highly reactive sites to enhance the photocatalytic activity toward the CIP degradation in aqueous solutions [28,29].

**Figure 2:**
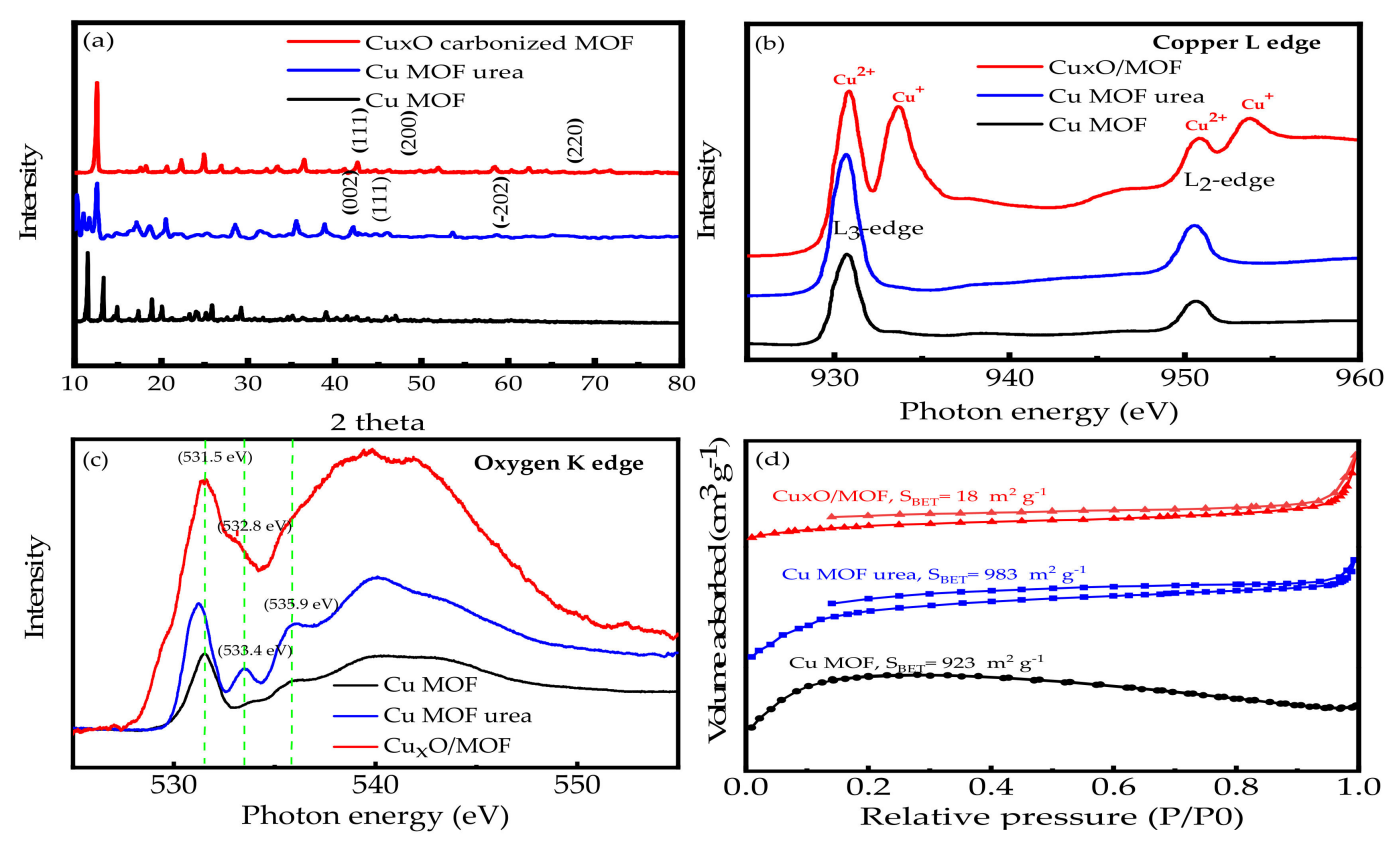
(**a**) XRD patterns and, (**b**) copper L-edge and (**c**) oxygen K edge XAS spectra and (**d**) N_2_ adsorption-desorption curves of the surface area of Cu-MOF, Cu-MOF-urea, and Cu_x_O/MOF after carbonization at 300 °C, in the presence of Ar/H_2_.

The X-ray diffractometer (XRD) patterns and X-ray absorption spectroscopy (XAS) spectra were further used to characterize the crystallinity and the fine structures of Cu-MOF, Cu-MOF-urea, and Cu_x_O/MOF. Figure 2a shows the XRD patterns of the MOF-based nanomaterials. The XRD pattern of the original Cu-MOF exhibits all of the diffraction peaks of Cu-MOF when BTC is used as the building unit [30]. Following the addition of urea for the hydrothermal treatment, the diffraction peaks at 35.5°, 38.5°, and 53.8° 2θ, correspond to the (002), (111), and ($\bar{2}$02) planes, respectively, which belong to the characteristic peaks of the monoclinic CuO (JCPDS No. 45-0937). The carbonization of Cu-MOF-urea at 300 °C changes the crystallinity of the Cu species, and the diffraction peaks at 36.4°, 43.2°, and 62.4° 2θ are clearly observed, which can be assigned as the (111), (200), and (220) lattice planes of cubic Cu_2_O (JCPDS No. 99-0041). Several studies have reported that Cu_2_O is more reactive than CuO [31], which indicates that the formation of the Cu_2_O-based MOF nanocomposite will be conducive to the enhanced photocatalytic activity of Cu_x_O/MOF.

The fine structures of the Cu_x_O/MOF nanocomposite were further investigated using XAS. The Cu L-edge spectra of Cu-MOF, Cu-MOF-urea, and Cu_x_O/MOF are shown in Figure 2b. The results obtained for Cu-MOF and Cu-MOF-urea are presented at 930.8 and 950.6 eV and could be associated with the Cu^2+^ species related to CuO [32]. The Cu L-edge of Cu_x_O/MOF has two significant peaks at 933.6 and 953.7 eV, representing an oxidation state of the Cu^+^ species, which is in agreement with the previous study related to Cu_2_O [32,33]. Figure 2c shows the XAS spectrum of Cu-MOF, showing an intense O K-edge peak at 531.5 eV, which is the typical CuO peak connected to the covalent oxygen-copper conduction band at O 2p with the Cu 3d electron. Moreover, the O K-edge spectrum of Cu_x_O/MOF exhibits the peak at 532.8 eV, due to the hybridization between the O 2p and Cu 3d states, which reported the same results with the Cu_2_O structure by Jiang et al. [34]. These results corroborate the formation of CuO and Cu_2_O on the Cu_x_O/MOF nanomaterial.

The specific surface area (S_BET_) of the MOF-based nanomaterials was determined. As shown in Figure 2d, the BET isotherm curves of both Cu-MOF and Cu-MOF-urea show a high surface area of 923 m^2^ g^−1^ and 983 m^2^ g^−1^, respectively, which exhibit typical Type I isotherms in the P/P0 range of 0.01–0.2 for micropores. There is also the presence of the hysteresis loops at the P/P0 range from 0.2 to 0.95, elaborating the mixture of the micro-meso pore structure originating from the square channels between the framework [35,36]. Therefore, Cu-MOF-urea through carbonization has slightly reduced the S_BET_ of the heterostructure to 18 m^2^ g^−1^, which is in agreement with the reported study [37]. The Cu_x_O/MOF decreases in S_BET_, which is probably due to the disintegration of the framework structure, leading to the occupation of the pores of Cu_x_O/MOF by the CuO/Cu_2_O nanocomposite. The pore size distributions were determined by applying the BJH calculation, based on the N_2_ adsorption isothermal. Figure S3 (Supplementary Data) shows the BJH isotherm curves of Cu-MOF, Cu-MOF-urea, and CuxO/MOF and shows the pore size of 1.99–2.11 nm, which is consistent with the reported results [38].

**Figure 3:**
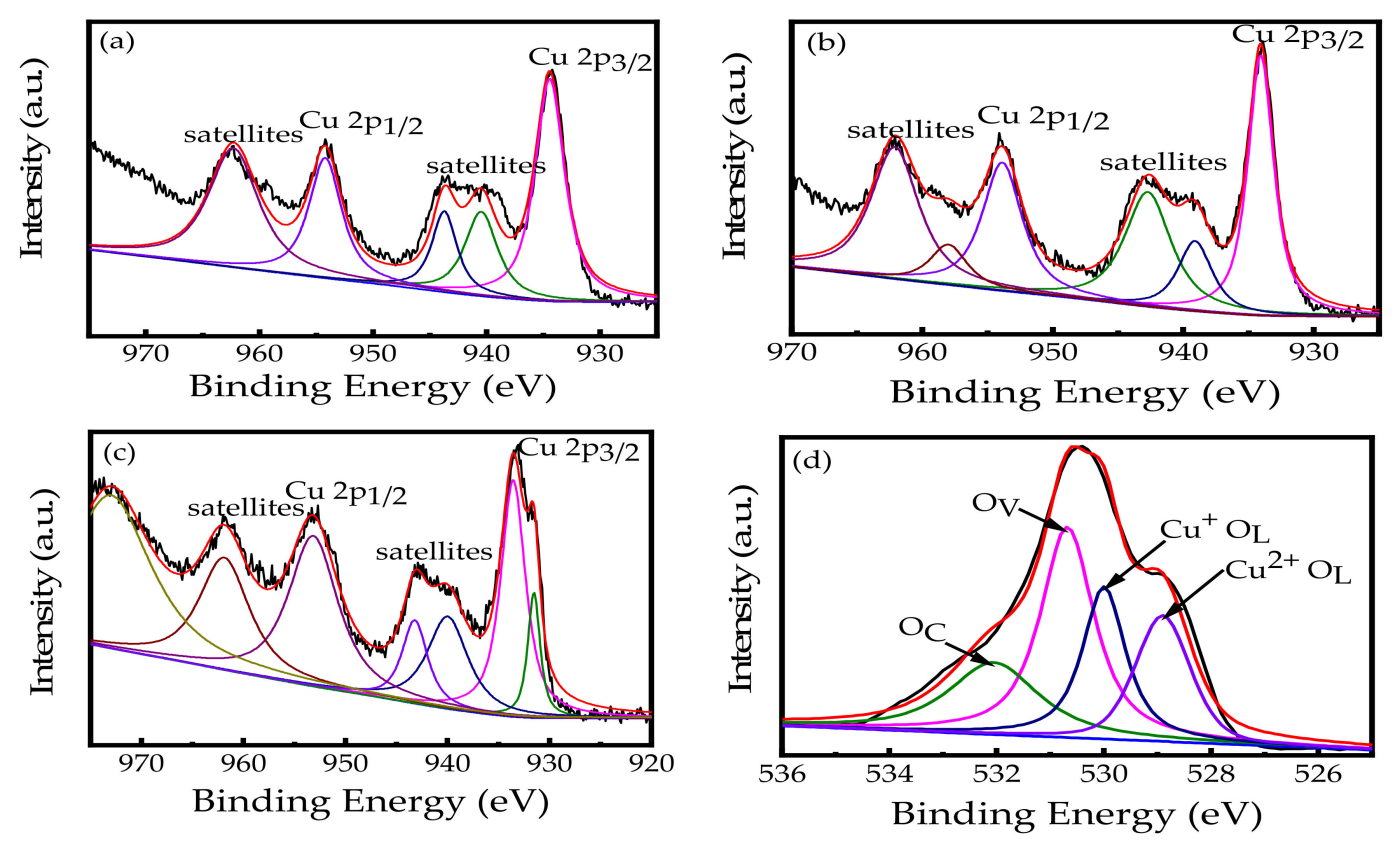
XPS spectra of Cu-MOF (**a**), Cu-MOF-urea (**b**) and CuxO/MOF (**c**) in the Cu region, and (**d**) O 1s of Cu_x_O/MOF.

The XPS data were further investigated to elucidate the surface chemistry of the Cu_x_O/MOF nanocomposite material. The peaks at 77, 122, 284, 530, and 933 eV, in the full scan spectrum of Cu_x_O/MOF, are primarily contributed from Cu 3p, Cu 3s, C 1s, O1s, and Cu 2p, respectively (Figure S4a, Supplementary Data). Following the peak deconvolution, Figs. 3a and 3b show the deconvoluted Cu spectra of Cu-MOF and Cu-MOF-urea, respectively. The binding energies at 934.4 and 954.4 eV are ascribed to the 2p_3/2_ and 2p_1/2_ peaks of divalent copper (Cu^2+^) [39,40], and the other satellite peaks corresponding to the electronic shake-up that was observed in Cu 2p of the three samples, which prove the existence of CuO on the cage surface [41]. The Cu 2p XPS spectra of the Cu_x_O/MOF nanocomposite possess another new Cu^+^ 2p_3/2_ peak at 931.6 eV (Figure 3c), confirming the formation of Cu_2_O after the carbonization at 300 °C [42]. Additionally, there are two peaks found at 283.3 and 287.1 eV in the C 1s spectrum (Figure S4 b (Supplementary Data)), which are assigned as sp^2^ carbon (C-C) and (C-O-C) [29]. Figure 3d shows the O 1s XPS spectra of Cu_x_O-MOF. The O1s curve is asymmetric and, it can be partitioned into four components through Gaussian fitting, including lattice oxygen of CuO (O_L_ (Cu^2+^), Cu_2_O (O_L_ (Cu^+^), oxygen vacancy (O_V_), and adsorbed oxygen (O_C_) [43]. This result confirms the formation of the Cu_2_O-CuO/MOF nanocomposite from Cu-MOF-urea after the carbonization at 300 °C, which is in agreement with the XRD and XAS results.

### 3.2. Adsorption and Photodegradation of CIP by Cu_x_O/MOF

Following the surface characterization, the photocatalytic activity of Cu_x_O/MOF was evaluated using CIP as the model compound. Figure S4 (Supplementary Data) shows the optical property of the MOF-based nanomaterials as a function of the bandgap. The calculated bandgaps for the as-prepared Cu-MOF, Cu-MOF-urea, and Cu_x_O/MOF, are 1.74, 2.92, and 2.45 eV, respectively. Interestingly, the photo-response of Cu_x_O/MOF occurs in the visible light region, which is close to the bandgap of Cu_2_O (2.2 eV) [40] signifying the plausible enhancement of its photocatalytic performance.

**Figure 4:**
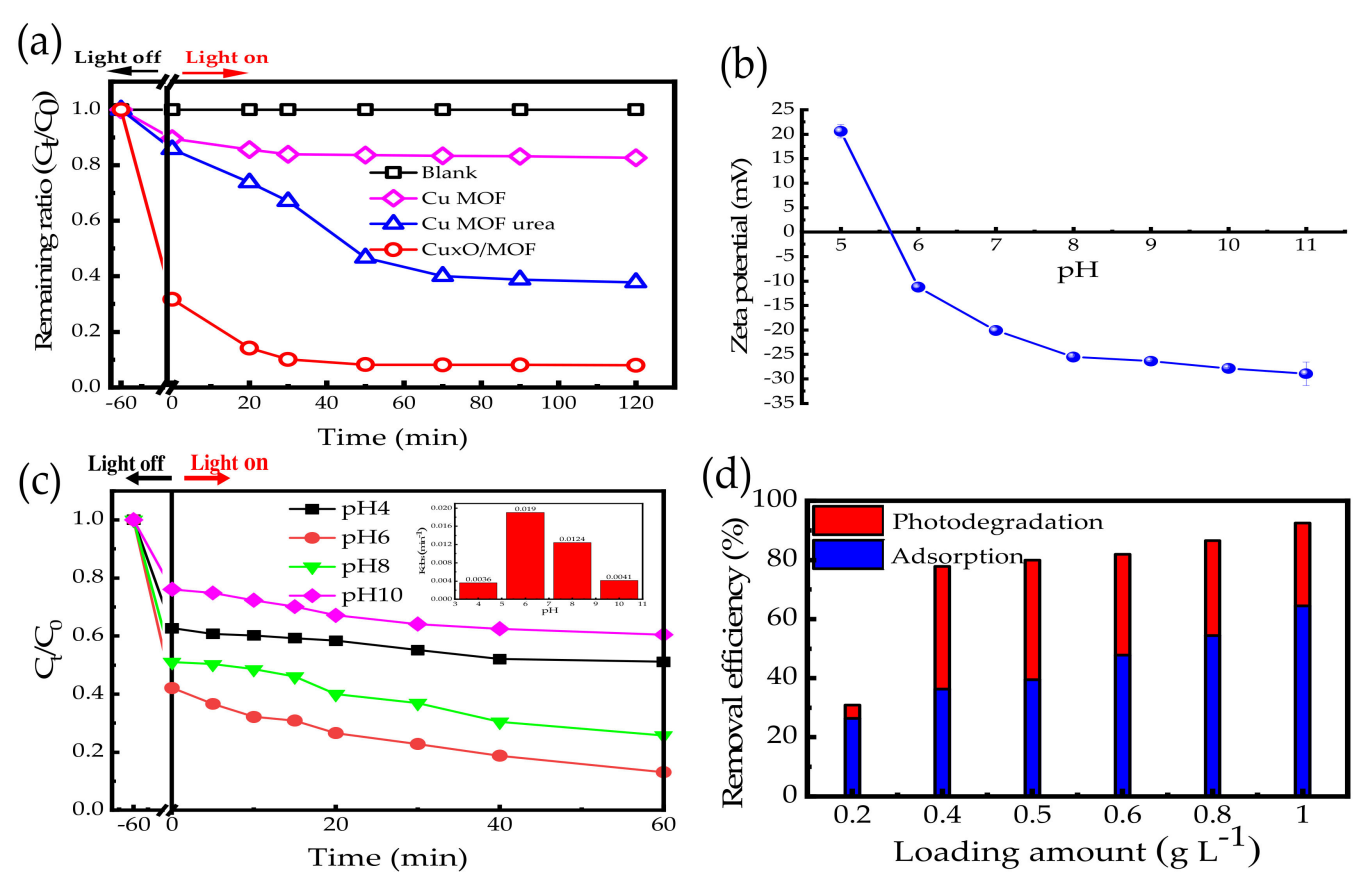
Photodegradation of 10 mg L^−1^ CIP over (**a**) 1.0 g L^−1^ of the MOF-based photocatalysts, and (**b**) zeta potential of Cu_x_O/MOF, (**c**) photodegradation efficiency of CIP by Cu_x_O/MOF as a function of the pH, ranging from 4 to 10, and (**d**) different loading amounts of Cu_x_O/MOF, ranging from 0.2 to 1.0 g L^−1^.

The removal of CIP by the MOF-based nanomaterials under visible light irradiation at 465 nm, is shown in Figure 4a. An insignificant reduction in the CIP concentration in the blank control was observed after 90 min of incubation, demonstrating the stability of CIP in the absence of the MOF photocatalysts and in the dark. Following the addition of Cu-MOF and Cu-MOF-urea as the photocatalysts, the removal efficiencies of 17 and 62% of the original CIP are observed after 90 min of irradiation. It is clear that only 10–15% of CIP is adsorbed within 60 min prior to the photoreaction, showing the low adsorption of CIP by Cu-MOF and Cu-MOF-urea. The addition of the Cu_x_O/MOF nanocomposite provides the highest removal efficiency of CIP, with a removal efficiency of 92% observed within 90 min. It is noteworthy to highlight that 68% of CIP is adsorbed by Cu_x_O/MOF, prior to the photodegradation. The relatively high adsorption percentage, in comparison with Cu-MOF and Cu-MOF-urea, is likely attributed to the carbonization procedure to convert the organic matter into carbon. In addition, the isoelectric point (pH_IEP_) of Cu_x_O/MOF is determined to be 5.6 (Figure 4b) and the pK_a_ values of CIP are at 6.2 and 8.6. This means that the Cu_x_O/MOF surface is negatively charged, while CIP is positively charged at the experimental pH value of 6.0. Therefore, the adsorbed amount of the positively charged CIP onto negatively charged Cu_x_O/MOF is high, due to the strong electrostatic interaction.

The next step was to apply a pseudo-first-order rate model to explore the photocatalytic degradation behavior of CIP over Cu_x_O/MOF. Following the subtraction of the adsorption part, the pseudo-first-order rate constants (k_obs_) of Cu-MOF, Cu-MOF-urea, and CuxO/MOF, towards the CIP photodegradation are 0.0017 (R^2^ = 0.992), 0.0083 (R^2^ = 0.986), and 0.0439 min^−1^ (R^2^ = 0.991), respectively. The excellent photocatalytic activity of Cu_x_O/MOF is probably due to the coexistence of CuO and Cu_2_O. It is noted that Cu_2_O is a visible-light-active semiconductor, which can give rise to the active sites surrounding the surface of Cu_x_O/MOF after irradiation, and subsequently results in the rapid CIP photodegradation in an aqueous solution.

The effect of the pH on the photocatalytic degradation of CIP by Cu_x_O/MOF, was assessed at pHs 4, 6, 8, and 10. As shown in Figure 4c, 51% and 60% of CIP are photodegraded at pHs 4 and 10 after 60 min of irradiation. The slight increase in pH improves the photodegradation efficiency of CIP to 75–85%, by using Cu_x_O/MOF at pH 6 and 8. The k_obs_ values for the CIP photodegradation at pH, ranging from 4–10, are 0.0036, 0.0190, 0.0124, and 0.0040 min^−1^, respectively (in Figure 4c inset). This result clearly indicates that pH 6 serves as an optimal condition for the photodegradation of CIP by Cu_x_O/MOF and is used for further experiments. In addition, the adsorption efficiency of CIP by Cu_x_O/MOF, in the dark, was observed at a different pH. As illustrated in Figure 4c in the dark region, the adsorption efficiency of CIP on Cu_x_O/MOF, is 38% under the acidic condition of pH 4 after 60 min of reaction, which is due to the revolting force between the positively charged Cu_x_O/MOF and cationic CIP on the Cu_x_O/MOF surface. When the pH increased to 6 and 8 adsorption efficiency of CIP increased by more than 50%, then decreases to 24% by increasing the pH to 10. Because the pKa1 and pK_a2_ of CIP are 6.09 and 8.74, respectively, it means that CIP is negatively charged (CIP^−^) at pH > 8.74, due to the carboxylic groups, while the zwitterionic CIP (CIP^±^) forms become dominant in the range of pH 6.09–8.74, due to the presence of the carboxylic and amino groups of piperazine [44,45]. It is noteworthy that the isoelectric point (pH_IEP_) value of Cu_x_O/MOF is 5.6, indicating that Cu_x_O/MOF is negatively charged at pH >5.6. Furthermore, when the pH increases to 10 with a pK_a2_ value > 8.74, the CIP species become anionic (CIP^−^), which are difficult to make CIP react with the negatively charged Cu_x_O/MOF, resulting in a decrease in the adsorption efficiency of CIP at high pH values.

Figure 4d shows the effect of the loading amount of Cu_x_O/MOF on the removal efficiency of CIP. The removal efficiency of CIP at 0.2 g L^−1^ Cu_x_O/MOF is 31%, which increases to 78–92% at 0.4–1.0 g L^−1^ Cu_x_O/MOF. The overall removal efficiency of CIP, as well as the adsorption efficiency, increases with the increase in the loading amount of Cu_x_O/MOF from 0.2 to 1.0 g L^−1^. The increment of the adsorption efficiency of CIP at a high Cu_x_O/MOF concentration is attributed to the electrostatic attraction between the positively charged CIP and the negatively charged Cu_x_O/MOF at pH 6. It is interesting to note that the photodegradation efficiency of CIP decreases from 40–42% at 0.4–0.5 g L^−1^ Cu_x_O/MOF to 28% at 1.0 g L^−1^ Cu_x_O/MOF, showing that the initial CIP concentration may play a crucial role in influencing the photocatalytic activity of Cu_x_O/MOF toward the CIP degradation.

### 3.3. Effect of the Initial CIP Concentration

**Figure 5:**
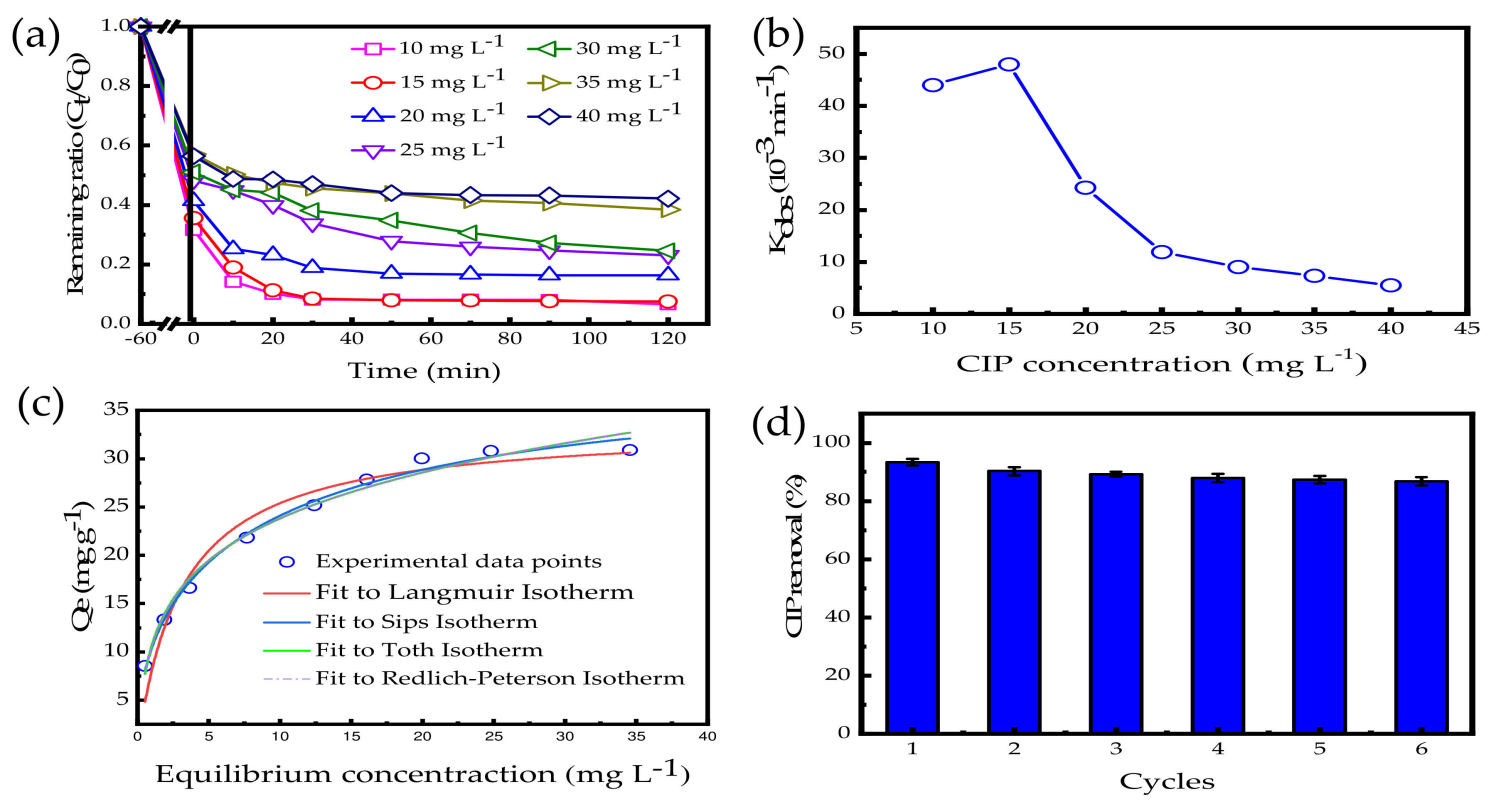
(**a**) The overall removal efficiency and (**b**) rate constants for the CIP photodegradation over 0.5 g L^−1^ Cu_x_O/MOF as a function of the initial CIP concentrations at 10–40 mg L^−1^, (**c**) adsorption isotherm of CIP with the fitted Langmuir, Sips, Toth, and Redlich-Peterson model, and (**d**) recyclability of Cu_x_O/MOF towards the CIP decomposition under visible light irradiation.

The results in Figure 5a show the effect of the initial CIP concentration on the removal efficiency of CIP over Cu_x_O/MOF. The initial concentrations of CIP used were 10–40 mg L^−1^, and the amount of Cu_x_O/MOF was 0.5 g L^−1^. The overall removal efficiency of CIP at 10–15 mg L^−1^ is greater than 90% after 30 min of reaction. However, the removal efficiency of CIP decreases with an increase in the CIP concentration between 20 and 40 mg L^−1^. The k_obs_ value for the CIP photodegradation increases slightly from 0.044 min^−1^ at 10 mg L^−1^ to 0.048 min^−1^ (r^2^ = 0.975) at 15 mg L^−1^, and then decreases rapidly to 0.0063 min^−1^ (r^2^ = 0.973) at 40 mg L^−1^ (Figure 5b).

Table 1 shows the comparison of the rate constant of the CIP photodegradation over Cu_x_O/MOF with other reported photocatalysts. A range of photocatalysts, including BiO_2_Br, WO_3_, AgBr, and MoS_2_ based nanocomposites, have been used as the effective photocatalysts for the CIP photodegradation under visible light irradiation conditions, and the k_obs_ values are in the range of 0.0094–0.0280 min^−1^ [46,47,48,49]. In this study, the as-prepared Cu_x_O/MOF nanocomposite exhibits the highest k_obs_ value for the CIP degradation in comparison with other commonly researched photocatalysts. The high removal efficiency and reaction rate of Cu_x_O/MOF can be attributed to the production of the homogeneous CuO-Cu_2_O nanoparticles onto the surface of the C-skeleton during the carbonization in H_2_/Ar. The produced CuO-Cu_2_O nanoparticles with carbon coating enhance the photocatalytic and adsorption capacities of Cu_x_O/MOF, corroborating that Cu_x_O/MOF is a superior nanomaterial that can eliminate CIP by both adsorption and visible-light-driven photodegradation.

The adsorption of CIP by the carbonized Cu_x_O/MOF also contributes 28–64% to the CIP removal. Therefore, the adsorption capacity of Cu_x_O/MOF was further examined under dark conditions at room temperature. Figure S6 (Supplementary Data) shows the change in the concentration of 10–40 mg L^−1^ CIP in the presence of 0.5 g L^−1^ Cu_x_O/MOF in the dark. It is shown that Cu_x_O/MOF is an effective adsorbent, and various concentrations of CIP decrease rapidly during the first 5 min and reaches the equilibrium after 40 min. Figure 5c shows the adsorption isotherm of CIP by Cu_x_O/MOF. Although the adsorption isotherm can be well-fitted by both Langmuir, Freundlich, Sips, Toth and Redlich–Peterson isotherm models, the Sips isotherm shows a higher correlation coefficient (R^2^ = 0.9893) than that of Freundlich, Langmuir, Toth, and Redlich–Peterson (R^2^ = 0.9487−0.9791). The model considered and the nonlinear regression parameters of each isotherm model are shown in Table S1. Therefore, Sips was the most suitable model to describe the CIP adsorption on CuxO/MOF, and the Langmuir isotherm is used to quantify the adsorption capacity of Cu_x_O/MOF. The Langmuir isotherm model can accurately describe the adsorption of CIP by Cu_x_O/MOF (Figure 5c). The maximum CIP adsorption capacity (q_max_) and affinity coefficient (K) are calculated to be 33.4 mg g^−1^ and 0.316 L mg^−1^, respectively, showing that CIP is relatively easy to be adsorbed onto the Cu_x_O/MOF surface. The Sips model is based on other assumptions that each adsorption site can interact with the heterogeneous adsorbent surface processes [52]. However, it was observed that the Toth and Redlich–Peterson models showed a similar of fitness for the CIP adsorption on CuxO/MOF. This might cause the adsorption of CIP to decrease linearly with the coverage of the adsorption sites and the adsorbent surface is closely homogeneous for CIP [52,53].

The longevity and reusability of Cu_x_O/MOF were further evaluated by the repeated injection of 15 mg L^−1^ of CIP into the batches containing 0.5 g L^−1^ Cu_x_O/MOF. Figure 5d shows the CIP degradation efficiency by Cu_x_O/MOF under six consecutive cycles. The 15 mg L^−1^ CIP was re-injected into the batches at the end of each reaction experiment. The overall removal efficiency of CIP was maintained stably in the range of 93–87% after six cycles. This result also signifies the adsorbed CIP can be photodegraded effectively under the visible light irradiation. Given that the decline in the removal rate was approximately 5% over six cycles, the consistent stability of Cu_x_O/MOF was considered acceptable for this study. The slight decrease in the removal efficiency of CIP might be due to the competition of active sites with the intermediates produced during the photodegradation process [45]. Furthermore, the increased active sites could be added activators of H_2_O_2_ or peroxymonosulfate (PMS) for the cycling process to maintain a high photodegradation efficiency.

### 3.4. Possible Mechanism for the Enhanced Photocatalytic Activity of Cu_x_O/MOF

**Figure 6:**
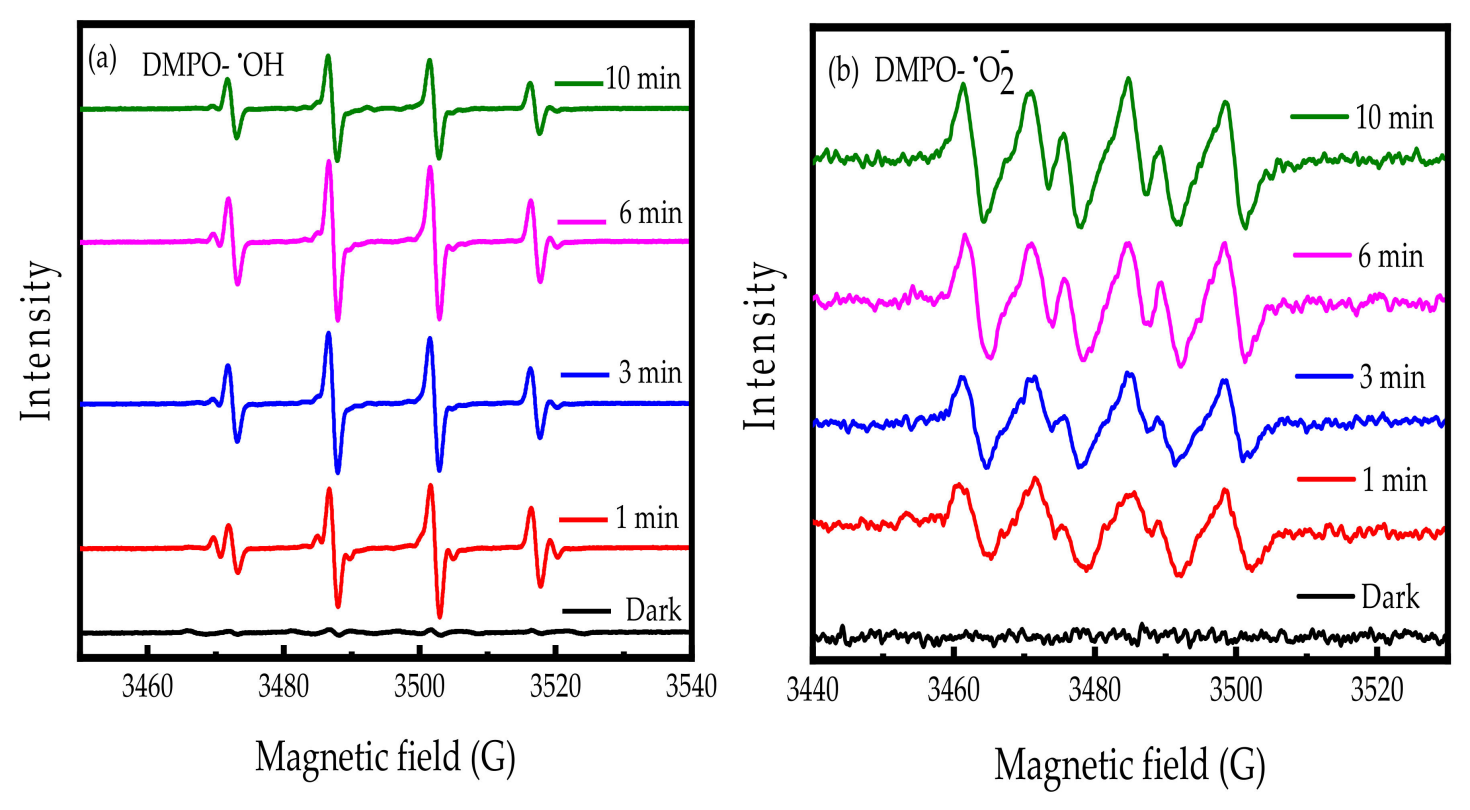
EPR spectra of the radical adducts trapped by (**a**) DMPO-OH in an aqueous solution, and (**b**) DMPO-O_2_^−^ in methanol in the Cu_x_O/MOF system.

In this study, the Cu_x_O/MOF nanocomposite exhibits an excellent overall removal efficiency and high photocatalytic activity toward the CIP degradation. To further investigate the photocatalytic mechanism for the CIP photodegradation over Cu_x_O/MOF, an EPR analysis using DMPO as the trapping agent is carried out, to identify the generation of the OH radicals in an aqueous solution and O_2_^−^ in methanol under visible light irradiation conditions [54]. The EPR spectra show that no signal is detected under the dark condition (Figure 6a). In contrast, strong signals with a peak intensity of 1:2:2:1, which belong to the OH radicals, are detected when Cu_x_O/MOF is irradiated with the visible light for 1–10 min [55]. Additionally, the DMPO-O_2_^−^ signals in the methanol solution are shown in Figure 6b. A 6-line signal in the Cu_x_O/MOF spectra is clearly observed, and the peak intensity increases as the irradiation time increases from 1 to 10 min. These results clearly indicate the generation of OH and O_2_^−^ radicals from Cu_x_O/MOF after irradiation with 465 nm visible light, which can significantly enhance the photodegradation efficiency of CIP over Cu_x_O/MOF.

**Figure 7:**
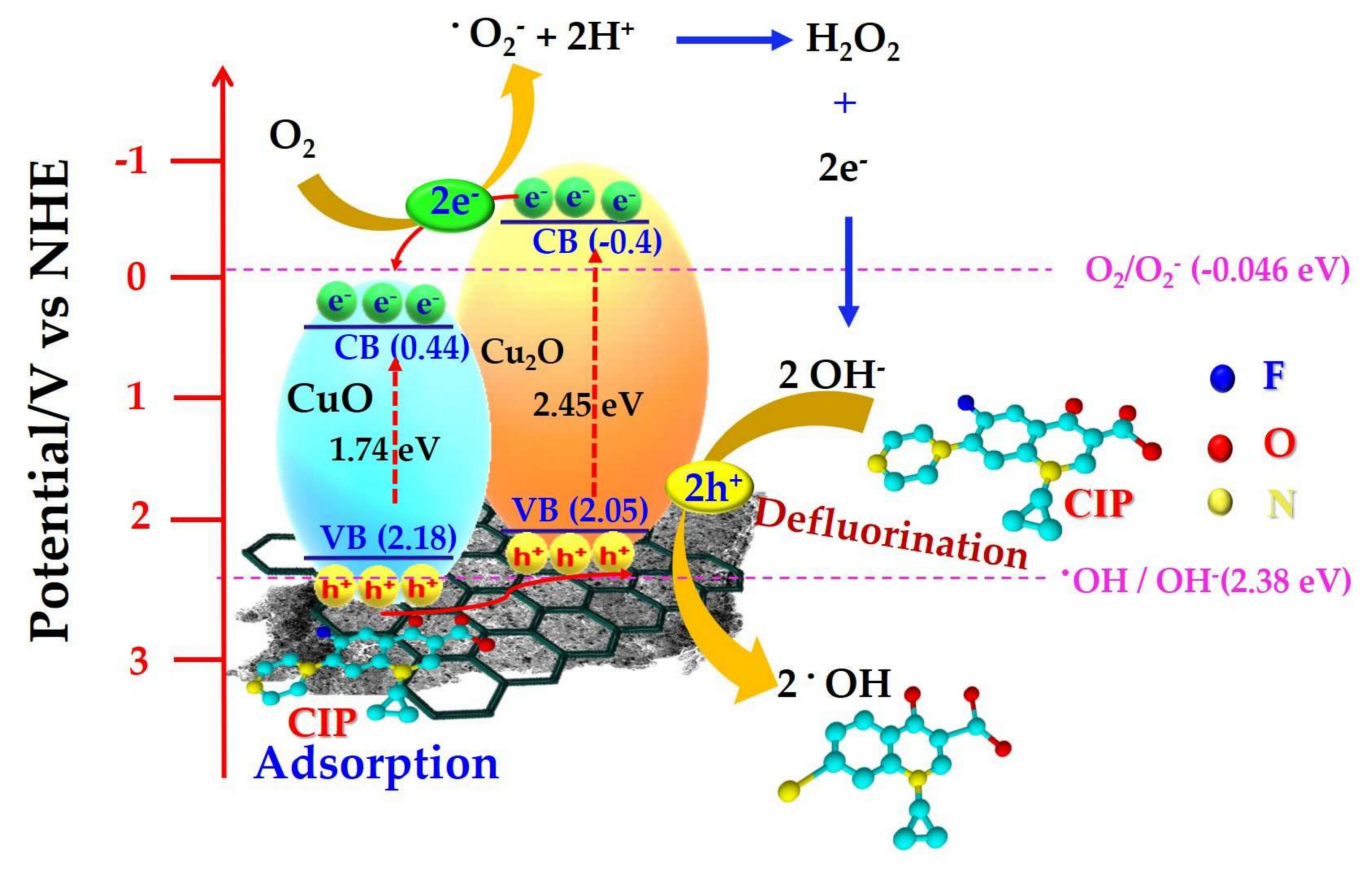
The proposed mechanism for the enhanced CIP photocatalytic activity using mixed phase Cu_x_O/MOF under visible light irradiation.

In this study, the carbonized MOF can serve as the adsorbent to remove CIP, as well as the support for CuO-Cu_2_O nanoparticles’ deposition, while CuO-Cu_2_O acts as the photocatalyst to produce the reactive oxygen species (ROS) for the effective photodegradation of CIP. It is interesting to note that the bandgap ($E_{g}$) is the bandgap of the monoclinic CuO structure (1.74 eV) in Cu-MOF and the cubic structure of Cu_2_O (2.45 eV) in Cu_x_O/MOF, determined from the UV-visible spectra, are 1.7 and 2.45 eV, respectively (Figure S5). Moreover, the potential of the valance band (E_VB_) of CuO and Cu_2_O, which can be calculated from a (our) previous study, are 2.18 and 2.05 V, respectively. Therefore, the possible visible-light-responsive photocatalytic mechanism for the CIP degradation over Cu_x_O/MOF is shown in Figure 7. Following the irradiation with the visible light at a wavelength < 460 nm, both CuO and Cu_2_O can generate the hole-electron pairs. Then, the electrons from the conduction band (CB) of Cu_2_O would likely move to the CB of CuO. At the same time, holes (h^+^) in the VB of CuO could energetically favorably transfer to the VB of Cu_2_O. Therefore, the CuO-Cu_2_O-C structure of Cu_x_O/MOF can effectively enhance the interfacial charge transfer efficiency, leading to the effective separation of h^+^ and e^−^. It is demonstrated that the photo-excited e^−^ in the CB of Cu_2_O can react with the surface absorbed O_2_ to generate the O_2_^–^ radicals because of the higher CB position (−0.4 V), in comparison with the reduction potential of O_2_^−^ (E^o^_O2/O2−_ = −0.046 V) [56]. The produced ROS can further react with e^−^ to generate H_2_O_2_ and OH [57]. Moreover, the carbonized MOF can protect Cu_2_O from oxidation caused by the nonselective active species, such as h^+^ and O and [29], therefore, it maintains the long-term photocatalytic activity of the multi-phase CuO-Cu_2_O-C structure of Cu_x_O/MOF.

### 3.5. Possible Photodegradation Pathway of CIP by Cu_x_O/MOF

To understand the possible photodegradation pathway of CIP, a liquid-chromatography mass spectrometer (LC-MS) was used to identify the intermediates produced from the photodegradation of CIP [45] over Cu_x_O/MOF. The LC-MS spectra of intermediates produced from the visible-light-driven photodegradation of CIP over Cu_x_O/MOF are shown in Figure S7 (Supplementary Data), and the molecular weight and structure of the intermediates are summarized in Table S2 (Supplementary Data).

**Figure 8:**
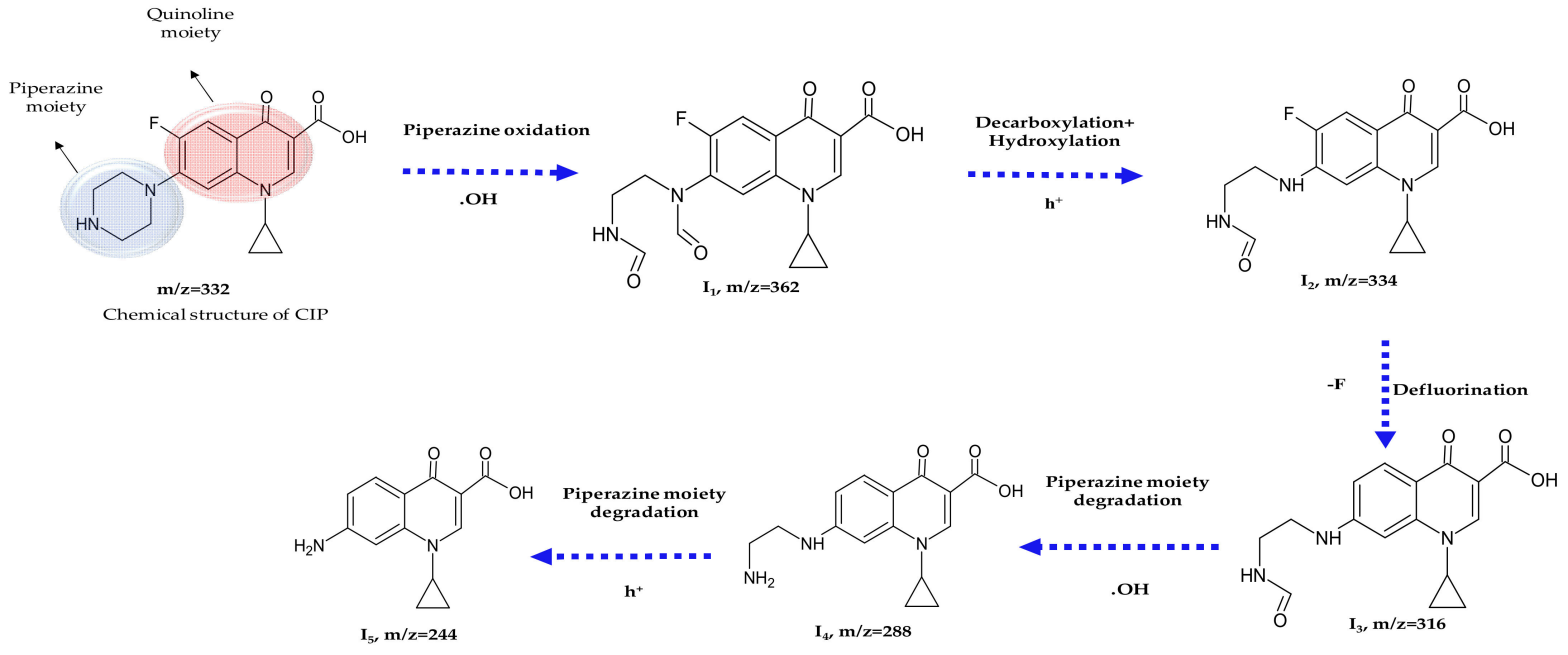
Possible pathway for the CIP photodegradation.

Figure 8 describes the possible degradation pathway of the CIP photodegradation. The reactions involve decarboxylation, piperazine oxidation, and defluorination, due to the presence of the carboxylic group and the strong electron-withdrawing fluorine substituents in CIP [58]. The inductive effect of the piperazine ring leads to the attack by the OH radicals, and results in the production of a dialdehyde derivative (I_1_) with *m*/*z* of 362. The further oxidation between the OH and C-N bonds results in the decarboxylation to produce two isomers of the mono-aldehyde intermediate (I_2_) with *m*/*z* of 334. A similar reaction between piperazine and the OH radicals has been reported when the ZnO-Ag_2_O/g-C_3_N_4_ and TiO_2_ photocatalysts were used for the CIP photodegradation [59,60]. The intermediate I_3_ with *m*/*z* of 316 is formed from the reductive defluorination of I_2_, which concurs with the reported literature [61,62]. Similarly, the intermediate I_4_ with *m*/*z* of 288 is from the decarboxylation of I_3_. The detection of the intermediate I_5_ can be ascribed to the loss of the -C_2_H_5_N fragment of I_4,_ due to the photo-oxidation hole (h^+^).

Several studies have reported the photocatalytic degradation of CIP in the presence of a photo-generated hole (h^+^) and the OH radicals. In general, the hole (h^+^) and OH radicals are most likely to attack the piperazine ring, in order to undergo the reductive defluorination for the CIP photodegradation over a broad variety of photocatalysts, including Bi_2_O_3_/(BiO)_2_CO_3_ [61]. TiO_2_ [63] Bi_2_WO_6_ [64], and gC_3_N_4_/TiO_2_/HNT [65] CIP can therefore be detoxified through the photocatalytic oxidation, hydroxyl radical reaction, photo-hole (h^+^) oxidation, and reductive defluorination. In this study, the possible degradation pathway of the CIP photodegradation follows the route: CIP → I_1_ (*m*/*z* 362) → I_2_ (*m*/*z* 334) → I_3_ (*m*/*z* 316) → I_4_ (*m*/*z* 288) → I_5_ (1-cyclopropyl 4-oxo 7-amino 1,4-dihydroquinoline 3-carboxylic acid, *m*/*z* = 244). A previous study used Fenton reagent to decompose CIP, and the end product after the Fenton reaction is similar to the result of this study (I_5_) [58]. Moreover, the toxicity of the end-product was confirmed to be low after the respirometric toxicity assessment [58]. This result indicates that Cu_x_O/MOF is not a concern to the environment and is a recyclable nanomaterial, which can serve as the dual function for the adsorbent, as well as a visible-light-driven photocatalyst for the effective CIP degradation in aqueous solutions.

## 4. Conclusions

In this study, a novel Cu_x_O/MOF nanocomposite consisting of a CuO-Cu_2_O-carbon structure was successfully synthesized for both the CIP adsorption and photodegradation. The stable chemical structure of CIP makes removing it from the water matrix difficult. Therefore, developing a novel and highly effective multifunctional technique for degrading CIP in water is urgently needed for the protection of water resources. Following the carbonization of Cu-MOF at 300 °C, in the presence of urea and 5% H_2_/Ar, Cu-MOF decomposes into an irregular shape, and the Cu ions have been converted to Cu_x_O nanoparticles, and then deposit onto the C-skeleton of MOF. The Cu_x_O nanoparticles exhibit a narrow distribution with an average lateral size of 9.2 ± 2.1 nm. The XAS and XPS analyses confirm that the existence of the CuO-Cu_2_O-carbon structure in the Cu_x_O/MOF nanocomposite, provides highly sensitive and active sites for the adsorption and photocatalytic degradation of CIP from aqueous solutions. Cu_x_O/MOF exhibits an excellent adsorption capacity of 34.5 mg g^−1^ for the CIP removal. Moreover, a superior photocatalytic activity of Cu_x_O/MOF toward the CIP photodegradation with a constant rate of 0.048 min^−1^, is also observed under visible light irradiation. The photogenerated holes (h^+^) and the ROS, such as O_2_^−^ and OH radicals, are the key reactive species for the CIP photodegradation, in terms of decarboxylation, piperazine oxidation, and defluorination. Moreover, Cu_x_O/MOF can be recycled at least six times without an appreciable loss of photoactivity toward the CIP degradation. However, further research is required to establish the full recyclability under real-world conditions.

The results obtained in this study clearly demonstrate the potential of the Cu_x_O/MOF nanocomposite for treating effluents containing CIP. The material has a dual-functionality of serving as an adsorbent and as a visible-light-driven photocatalyst that can rapidly degrade CIP and other emerging pollutants in aquatic environments. To establish the applicability under real world conditions, further research is needed to consider the operational factors, including the matrix of natural water and effluent samples which may influence the performance. Further research is also needed to establish the possible leaching and the potential contamination of the treated water and other potential adverse environmental behaviours and the risks of antibiotics.

## Figures and Tables

**Table 1:** Comparison of the CIP photodegradation kinetic over Cu_x_O-MOF with other reported visible-light-driven photocatalysts.

Photocatalyst	Dosage (g L^−1^)	Light Source	k_obs_ (min^−1^)	Ref.
BiO_2_Br/0.5Mn-PANI	-	Sunlight	0.0280	[46]
5%WO_3_/g-C_3_N_4_	1.0	Solar light	0.0256	[47]
0.5 AgBr/Ag_2_MoO_4_@ZnO	0.8	420 nm	0.0094	[48]
WO_3_/Bi_2_WO_6_	0.4	420 nm	0.0133	[50]
MoS_2_/ZIF8	0.4	420 nm	0.0099	[51]
Cu_x_O/MOF	0.5	465 nm	0.0480	This study

BiO_2_Br/0.5Mn-PANI: BiOBr@polyaniline-MnO_2_; 5% CNT/PbBiO_2_Br: carbon nanotube/PbBiO_2_Br; MoS_2_/ZIF8: MoS_2_/zeolitic imidazolate framework.

## Data Availability

The data presented in this study are available on request from the corresponding author.

## Supplementary Materials

The following supporting information can be downloaded at: https://www.mdpi.com/article/10.3390/nano13020282/s1, Figure S1, SEM image of Cu-MOF-urea, Figure S2, SEM image of CuxO/MOF; Figure S3, BJH isotherm curves of pore size of Cu-MOF(a), Cu-MOF-urea(b), and CuxO/MOF(c) after carbonization at 300 °C in the presence of Ar/H2; Figure S4, XPS analysis of the CuxO/MOF (a) full scan, (b) C 1s peaks; Figure S5, The Kubelka-Munk plots of the as-prepared materials; Figure S6, Adsorption of CIP in the range of concentration from10 to 40 mg L-1 in the presence of 0.5 g L-1 CuxO/MOF in the dark; Figure S7, The MS spectra of intermediates produced from the CIP photodegradation by CuxO/MOF at different incubation period; Table S1, Isotherm model constant and nonliner regression parameters for fit of CIP adsorption; Table S2, CIP degradation pathway of Intermediates information.
