# Supplementary material for: Enhanced Visible-Light-Responsive Photocatalytic Degradation of Ciprofloxacin by the CuxO/Metal-Organic Framework Hybrid Nanocomposite
Source: Nanomaterials (Basel). 2023 Jan 9;13(2):282. doi: 10.3390/nano13020282 (PMC9864703; doi:10.3390/nano13020282)
Supplement: Supplementary file 1 [file nanomaterials-13-00282-s001.zip › nanomaterials-2129063-supplementary.pdf]

nanomaterials

Supplementary information

Enhanced Visible-Light-Responsive Photocatalytic Degradation of Ciprofloxacin by the Cu_xO/Metal-Organic Framework Hybrid Nanocomposite

Cheng-Kuo Tsai [1,2,*], Ching-Hsuan Huang [3], Jao-Jia Horng [1,2], Hui Lin Ong [4,5,*] and Ruey-An Doong [6]

[1] Department of Safety Health and Environmental Engineering, National Yunlin University of Science and Technology, Douliou 64002, Taiwan
[2] Emergency Response Information Center, National Yunlin University of Science and Technology, Douliou 64002, Taiwan
[3] Institute of Environmental Engineering, National Yang Ming Chiao Tung University, Hsinchu 30010, Taiwan
[4] Faculty of Chemical Engineering Technology, Universiti Malaysia Perlis, Arau 02600, Malaysia
[5] Centre of Excellence for Biomass Utilization and Taiwan-Malaysia Innovation Centre for Clean Water and Sustainable Energy (WISE Centre), Universiti Malaysia Perlis (UniMAP), Jejawi 02600, Malaysia
[6] Institute of Analytical and Environmental Sciences, National Tsing Hua University, Hsinchu 30013, Taiwan
* Correspondence: cktsai@yuntech.edu.tw (C.-K.T.); hlong@unimap.edu.my (H.L.O.)

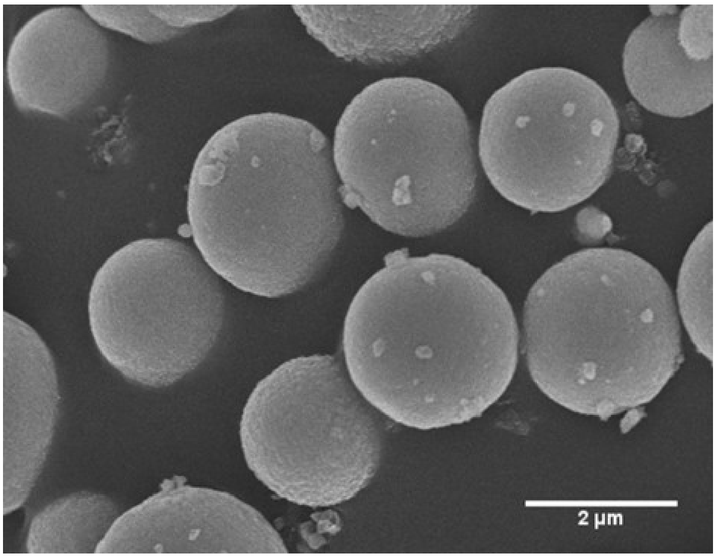

Figure S1. SEM image of Cu-MOF-urea.

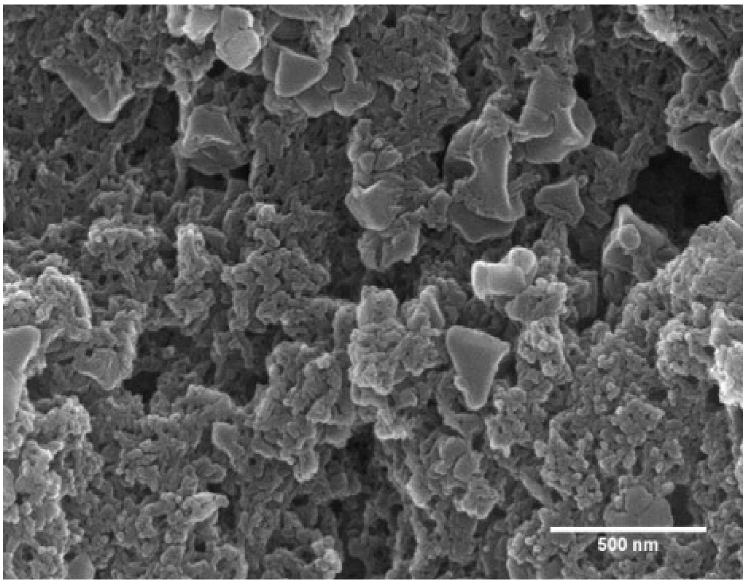

Figure S2. SEM image of Cu_xO/MOF.

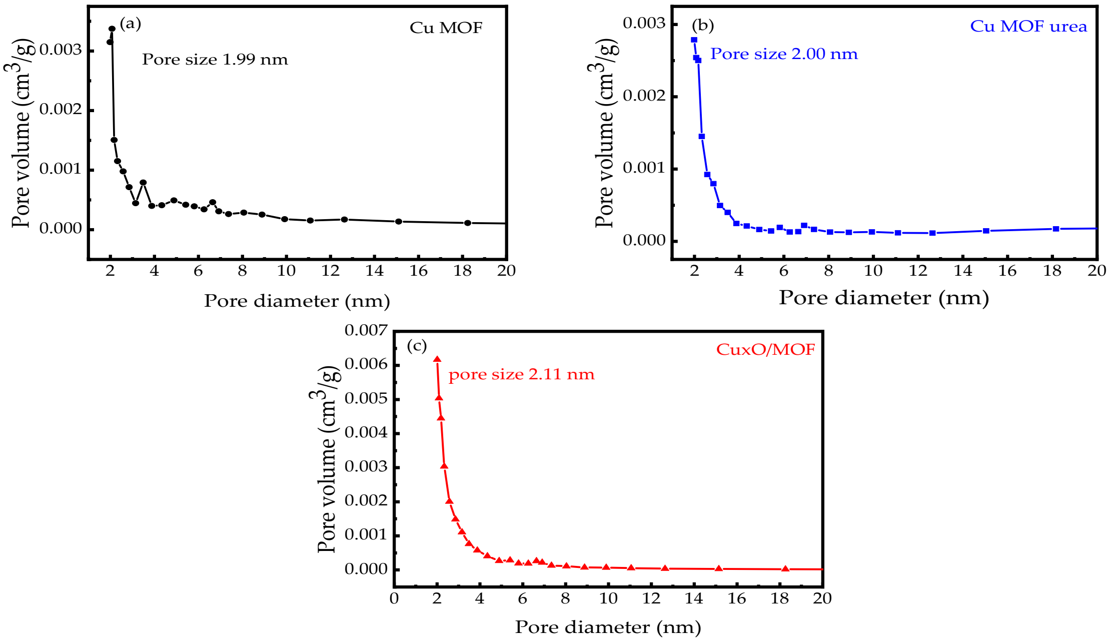

Figure S3. BJH isotherm curves of pore size of Cu-MOF(a), Cu-MOF-urea(b), and Cu_xO/MOF(c) after carbonization at 300 °C in the presence of Ar/H_2.

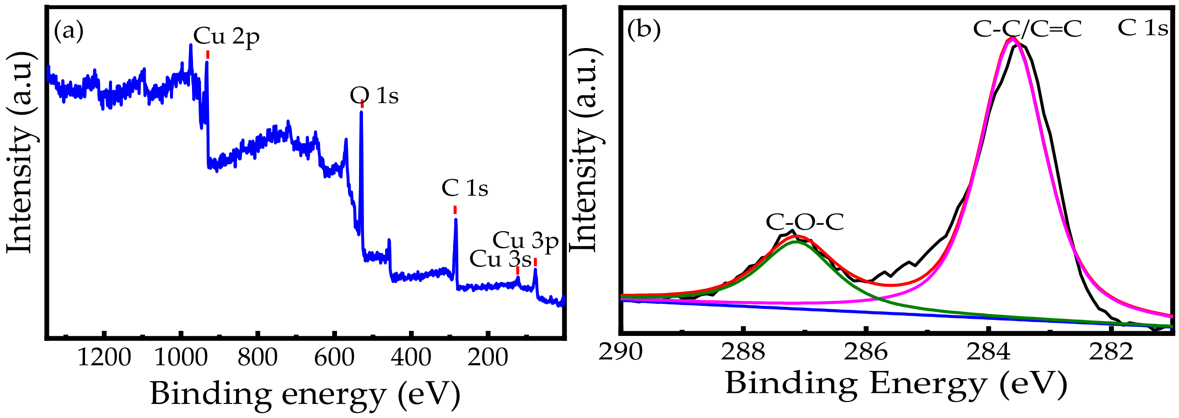

Figure S4. XPS analysis of the Cu_xO/MOF (a) full scan, (b) C 1s peaks.

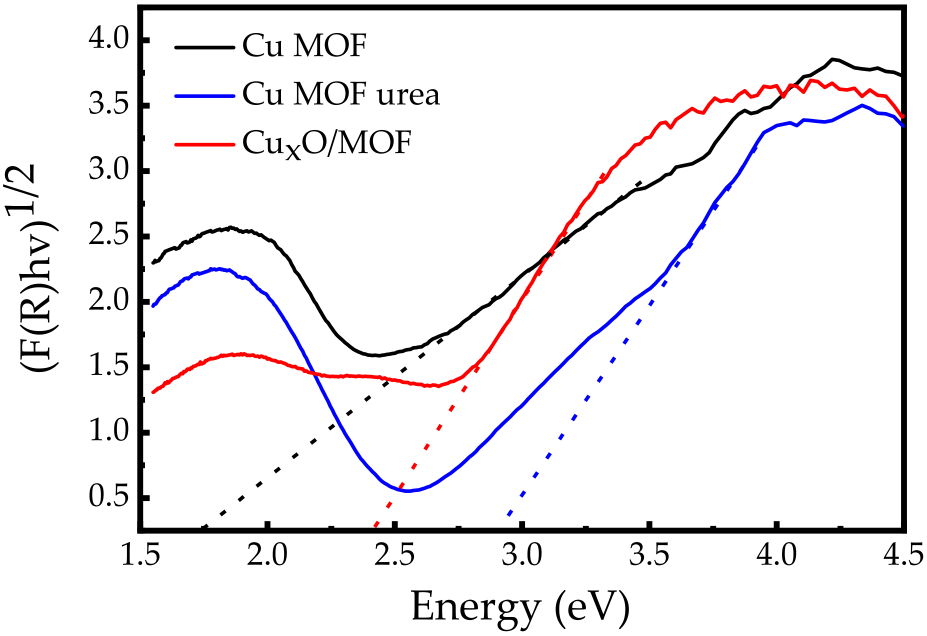

Figure S5. The Kubelka-Munk plots of the as-prepared materials.

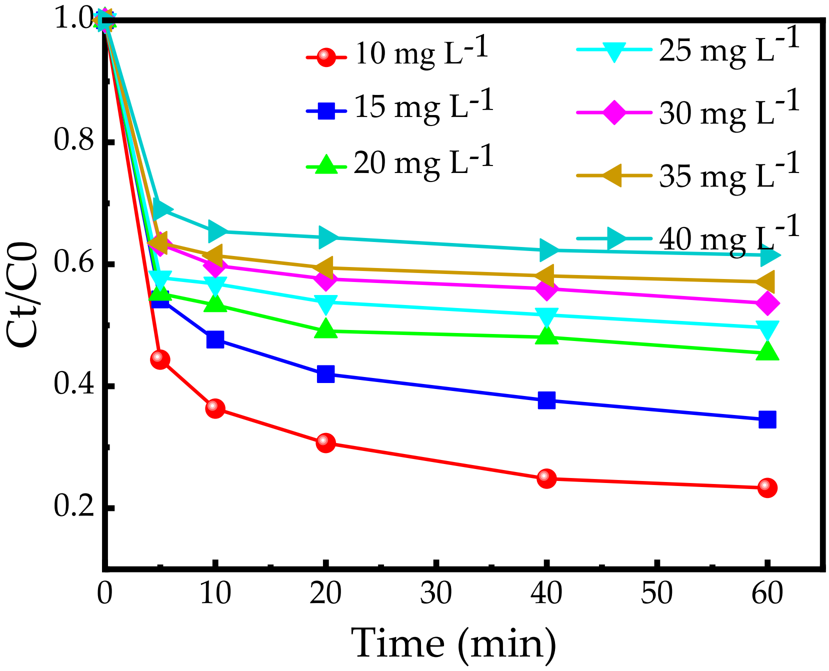

Figure S6. Adsorption of CIP in the range of concentration from10 to 40 mg L^{-1} in the presence of 0.5 g L^{-1} Cu_xO/MOF in the dark.

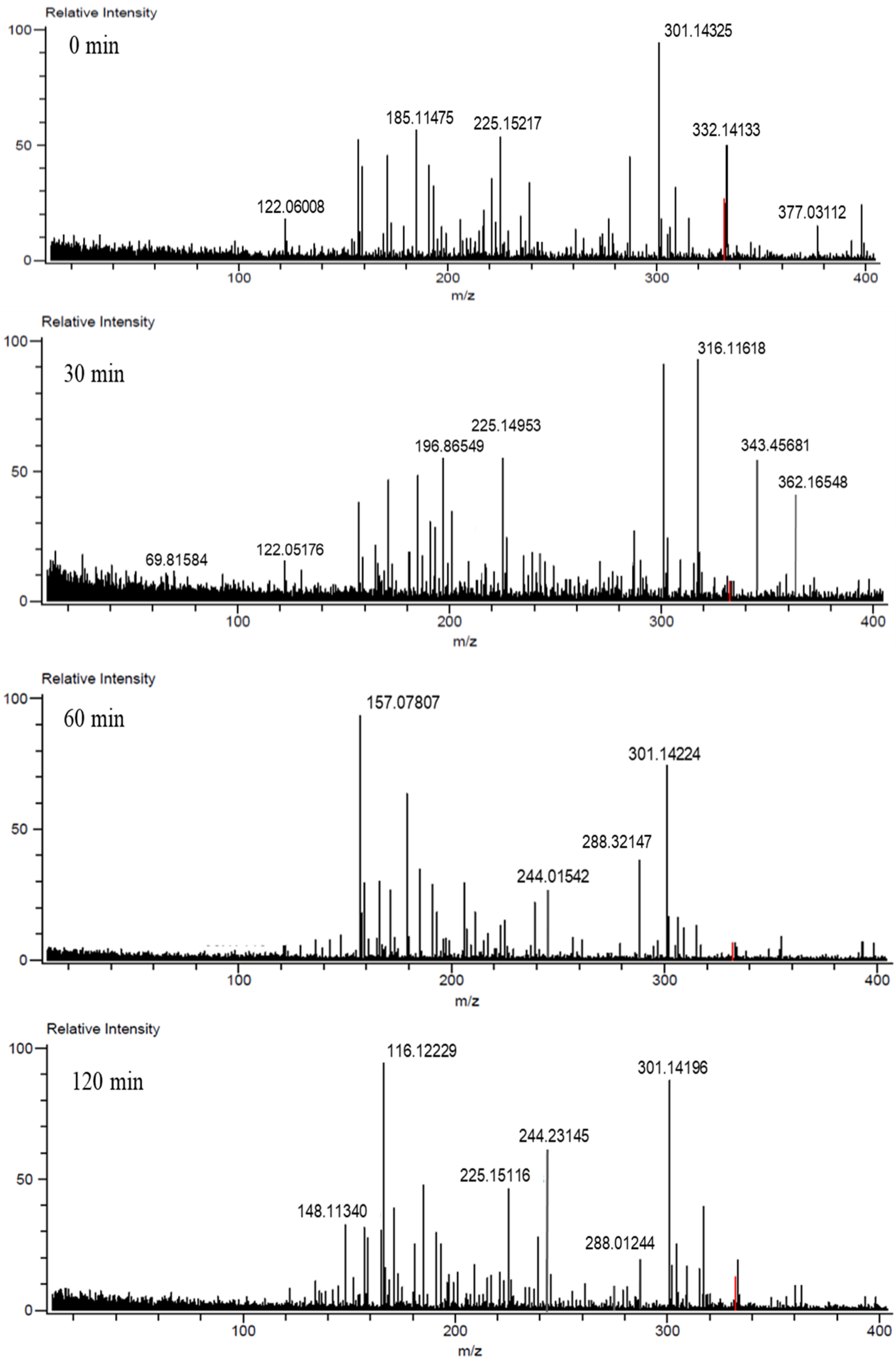

Figure S7. The MS spectra of intermediates produced from the CIP photodegradation by CuxO/MOF at different incubation period.

Table S1. Isotherm model constant and non-liner regression parameters for fit of CIP adsorption.

Isotherm models	Parameters	CIP
Langmuir	Qm (mg g^{-1})	33.411
	K_L	0.3163
	R^2	0.9487
Freundlich	Kf	2.90
	N	3.15
	R2	0.9691
Sips	Qm (mg g^{-1})	39.653
	K_S	0.045
	m	0.4262
	R^2	0.9893
Toth	Qm (mg g^{-1})	12.32
	K_T	2.765
	t	0.7799
	R^2	0.9791
Redlich-Peterson	K_R	34.03
	a	2.207
	b	0.779
	R^2	0.9791

Table S2. CIP degradation pathway of Intermediates information.

Product name	Molecular weight	Chemical formula and name
CIP	332	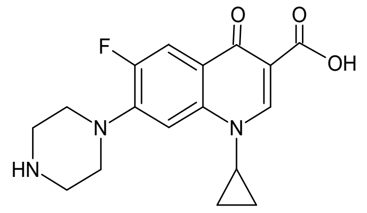 Chemical structure of CIP
I_1	362	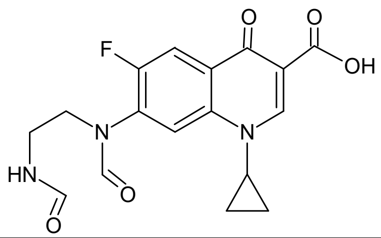

		1-cyclopropyl-6-fluoro-7-{formyl[2-(formylamino)ethyl]amino}-4-oxo-1,4-dihydroquinoline-3-carboxylic acid
I_2	343	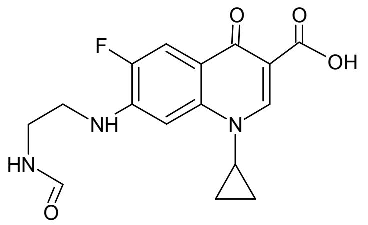 1-cyclopropyl-6-fluoro-7-{[2-(formylamino)ethyl]amino}-4-oxo-1,4-dihydroquinoline-3-carboxylic acid

Table S2, Continued

Product name	Molecular weight	Chemical formula
I_3	316	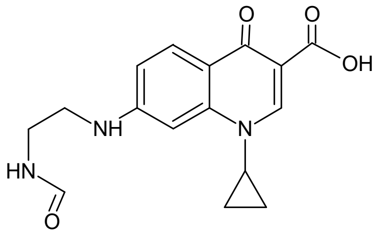 1-cyclopropyl-7-{[2-(formylamino)ethyl]amino}-4-oxo-1,4-dihydroquinoline-3-carboxylic acid
I_4	288	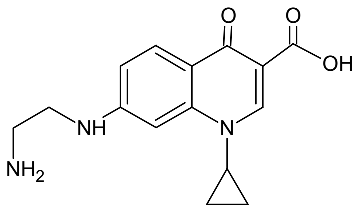 7-[(2-aminoethyl)amino]-1-cyclopropyl-4-oxo-1,4-dihydroquinoline-3-carboxylic acid
I_5	244	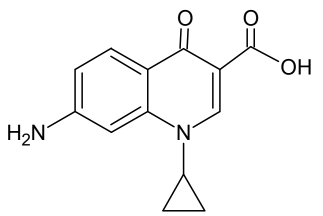 1-cyclopropyl 4-oxo 7-amino 1,4-dihydroquinoline 3-carboxylic acid
